# Short-Term Efficacy of High-Intensity Laser Therapy in Alleviating Pain in Patients with Knee Osteoarthritis: A Single-Blind Randomised Controlled Trial

**DOI:** 10.1155/2022/1319165

**Published:** 2022-10-21

**Authors:** Punpetch Siriratna, Chompoonuch Ratanasutiranont, Thongsuk Manissorn, Nonthalee Santiniyom, Waree Chira-Adisai

**Affiliations:** ^1^Department of Rehabilitation Medicine, Faculty of Medicine, Ramathibodi Hospital, Mahidol University, Bangkok, Thailand; ^2^Thailand National Sports University Sukhothai Campus, Faculty of Sports and Health Science, Sukhothai, Thailand

## Abstract

**Objectives:**

The aim of the study is to evaluate the efficacy of high-intensity laser therapy (HILT) on pain reduction in patients with knee osteoarthritis (OA).

**Methods:**

Forty-two patients diagnosed with primary knee OA, with a Kellgren–Lawrence classification of 2–4, were recruited into the study. The patients were randomly allocated to two groups: HILT and control. The intervention group received HILT (energy density of 22.39 J/cm2, 562.5 joule/session), while the control group received a sham laser, which was done 2–3 sessions per week for a total of 10 sessions. Both the groups also received the same conservative treatment. The main outcome measures were the visual analogue scale (VAS) and the modified Thai version of the Western Ontario and McMaster Universities Osteoarthritis Index (T-WOMAC) which were evaluated at baseline and immediately after treatment completion.

**Results:**

At the end of the study, the overall analysis showed a significant decrease in VAS and T-WOMAC scores in both the groups; a greater decrease in scores was found in the HILT group than in the control group (*p* < 0.001). The between-group comparison also showed a significant difference in VAS, but not in the T-WOMAC score, favouring HILT (*p* < 0.05).

**Conclusion:**

The HILT plus conservative treatment can help alleviate pain in patients with knee OA. The findings of the present study could be used in clinical practice to add HILT as another noninvasive treatment option for knee OA. This could be advantageous, particularly for individuals who are at high risk of surgery due to multiple comorbidities or older people. *Trial Registration*. This clinical trial registration was performed at Clinical.gov (NCT04889885).

## 1. Introduction

Knee osteoarthritis (OA) is one of the most common causes of knee pain worldwide. Osteoarthritic knee pain is caused by repetitive stress and mechanical loading that generates an inflammatory process within the knee joint, resulting in articular cartilage destruction and chondrocyte loss [1]. Prostaglandin *E*_2_ and metalloproteinases, the cartilage matrix-degrading enzymes, have also been found to play a significant role in this degrading process. Additionally, the repetitive loading results in transformation in the osteochondral junction as it will develop new vascular channels and innervation within this area. The vascular channels would aid in the transport of biochemical substances, such as cytokines, through the articular cartilage and subchondral bone [2]. Whereas, the neo-nerves would receive noxious stimuli from the knee joint, generating a pain signal that transmits through the nociceptive fibers as peripheral sensitization. When this process repeatedly occurs, it will reproduce afferent inputs to the dorsal horn of the spinal cord, leading to the wind-up phenomenon and eventually central sensitization [3]. As this vicious cycle occurs over time, patients with knee OA may experience difficulty in ambulation as well as functional deterioration and decreased health-related quality of life (HRQoL), especially those with severe knee OA [4, 5]. Previous evidence has also demonstrated that knee OA is associated with an increase in all-cause mortality and death from cardiovascular events [6]. In Thailand, the prevalence of knee OA has gradually increased over time, especially in the elderly population [7]. Thailand is currently moving towards an aged society; therefore, an increase in knee OA prevalence would contribute to the burden on the Thai healthcare system in the future.

In terms of general musculoskeletal pain management, conservative treatment remains the first line approach, which primarily consists of pharmacological and nonpharmacological treatments. A recent systematic review with network meta-analysis revealed that nonpharmacological treatments—for example, exercise, heat modality, and manual therapy—were efficacious for pain relief and reducing disability in musculoskeletal conditions, such as nonspecific low back pain [8]. Furthermore, another recent study found that complementary and alternative medicine like herbal extracts may also help to alleviate pain due to their antinociceptive and anesthetic effects [9]. The advantages of these nonpharmacological treatments are their positive effects on pain reduction and safety compared to the commonly used analgesics such as oral NSAIDs. For conservative treatment of knee OA, guidelines recommend land-based or water-based exercise, weight reduction, and cane use as the mainstay of treatment. Additionally, topical NSAIDs and intraarticular corticosteroid injections can also be used for short-term pain relief, whereas other medications, such as oral NSAIDs, opioids, and acetaminophen, should be used with caution due to the risk of potential adverse effects, especially in high-risk patients who have multiple comorbidities [10–12]. Other alternative treatment options include intraarticular hyaluronic acid, platelet-rich plasma, stem cells, or gene therapy. However, more research is still needed to demonstrate their beneficial effects and safety in knee OA [2].

Apart from the abovementioned conservative treatments, physical modalities could be used as a part of treatment in knee OA, especially for pain relief. Light amplification by stimulated emission of radiation (LASER) is a physical modality used to treat various musculoskeletal pain conditions. This is due to its ability to reduce pain, edema, and inflammation, and promote tissue healing [13–15]. Generally, there are two types of laser treatments classified by output power: low-level laser therapy (LLLT) and high-intensity laser therapy (HILT) that are commonly used in clinical practice. The LLLT has an output power of less than 500 mW. Previous studies have shown that the LLLT reduces inflammatory markers and the expression of proinflammatory cytokines, such as interleukin-1, interleukin-6, and prostaglandin *E*_2_ [14, 16]. Moreover, the LLLT could retard the loss of collagen type II, aggrecan, and transforming growth factor beta, which are essential for intraarticular cartilage function as well as the healing process [17]. Although current treatment guidelines do not recommend LLLT as an effective treatment for knee OA, a recent systematic review showed positive results for LLLT in terms of pain relief and reduced disability [18]. However, owing to the low output power of the LLLT, the depth of tissue penetration is limited. Additionally, treatment duration would be prolonged, especially for patients who require high-dose laser treatment.

For the past decade, HILT has been developed for the treatment of a wide range of musculoskeletal disorders [19]. In patients with knee OA, previous studies have shown positive effects of HILT in terms of pain relief, range of motion, and functional improvement over control [20–22]. Moreover, there is evidence that treatment with HILT is superior to LLLT in knee OA [23]. This superiority could be due to its high output power, which promotes deeper tissue penetration and releases a large amount of energy in a short period. Thus, it is a more effective treatment than LLLT. Although previous studies have shown positive results for HILT, a standardised treatment protocol for HILT in patients with knee OA has not been established. Moreover, a recent systematic review of HILT in the treatment of knee OA [24] reported significantly high heterogeneity, possibly due to differences in patient characteristics and laser treatment protocols in each study. Therefore, the main objective of this study was to compare the efficacy of another HILT protocol to sham laser plus conservative treatment in terms of pain reduction in patients with knee OA.

## 2. Materials and Methods

The present work is a randomised, single-blind, parallel-group study that aimed to compare the efficacy of HILT with that of sham laser plus conservative treatment. The trial was conducted in accordance with the Declaration of Helsinki and was approved by the Committee on Human Rights Related to Research involving Human Subjects, Faculty of Medicine, Ramathibodi Hospital, Mahidol University, Thailand (MURA 2016/242). The study was conducted at the outpatient clinic, rehabilitation medicine department from 1 June 2016 to 31 August 2016.

### 2.1. Participants

Forty-two participants with knee OA who fulfilled the eligibility criteria were enrolled in this study. All patients were evaluated by physiatrists including assessment of a baseline characteristics, history of knee OA, and physical examination. The inclusion criteria were as follows: (a) primary knee OA diagnosed on the basis of the American College of Rheumatology criteria 2016; (b) knee pain for at least 6 months with a visual analogue scale (VAS) score greater than 4; and (c) mild, moderate-to-severe knee OA, based on the Kellgren and Lawrence (KL) classification [25] which was graded and officially reported by radiologists. The exclusion criteria were as follows: (a) knee OA caused by other pathologies or secondary knee OA; (b) treatment with corticosteroid or hyaluronic injection into the knee joint within the past 6 months; (c) treatment with any other physical modalities at least 1 month before starting the trial; (d) a history of central and/or peripheral nervous system disorders, such as cerebrovascular accident (CVA), spinal cord injury, and peripheral neuropathy; (e) history of bleeding disorders, cancer, or deep vein thrombosis; (f) cognitive impairment or mental disorders; and (g) contraindication to laser therapy or sensitivity to light.

For randomisation, 42 participants who met the inclusion criteria were randomly allocated to two groups: HILT and sham laser plus conservative treatment. The randomisation process was concealed using sequentially numbered, opaque, and sealed envelopes. The participants did not know the treatment group to which they were assigned. However, the operator was aware of the group allocation. Written informed consent was obtained from all participants before starting the trial.

All participants were provided full information regarding the study and written informed consent was obtained before their first visit. Participants were encouraged to contact specialist staff if they had any queries or required troubleshooting during the study period. They were also allowed to discontinue participation from the study at any time that they felt uncomfortable.

### 2.2. Intervention

The participants received HILT using a Mphi laser device (ASA, Arcugnano, Italy) and a multiwave locked system (MLS). The MLS is a diode type, Nd: YAG, high-intensity laser combining two wavelengths of 808 nm (continuous emission) and 905 nm (pulsed emission) as a single pulse. This combination of the two wavelengths would attenuate the power of the treatment and promote more effective treatment with fewer side effects. The MLS can generate peak power of up to 25 W, frequency of 2,000 Hz, and a duty cycle of 50%, with spot diameter of 2 cm^2^ and spot area of a 3.14 cm^2^.

Regarding the laser treatment protocol in this study, the MLS was administered perpendicularly onto eight points around the knee joint based on a study by Hegedus et al. [26] The first six points were applied to the medial and lateral epicondyle of the femur, medial and lateral condyle of the tibia, and medial and lateral knee gaps in the supine position with 30° of knee flexion. The latter two points were applied to the medial edge of the biceps femoris and semitendinosus tendons in the prone position with 0° of knee extension (Supplemental Figure S1). Because there is no standardised recommended dosage of HILT for knee OA, the protocol used in this study was performed based on the World Association Laser for Therapy (WALT) for low-level laser therapy in knee OA (WALT), [27] and a systematic review of LLLT for pain from chronic joint disorders [28]. Therefore, the HILT was released at 70.31 Joules (J)/point with an energy density of 22.39 J/cm^2^ by using trigger point mode. The laser energy per session was 562.50 J and the duration of treatment was 8 minutes. The HILT was performed in 2–3 sessions per week for a total of 10 sessions over 4–5 weeks. All laser sessions were done by two physiotherapists blinded to the outcomes assessment.

In the control group, the participants received a sham laser using the same protocol as described in the intervention section. During each treatment session, the audio-beeping sound of the MLS machine was initiated to mimic the sound while the actual MLS machine was running. However, true laser emission was not released, so the participants were unaware of the treatment group allocation. For safety, goggles were prepared for all participants and physiotherapists during the treatment sessions to prevent retinal damage.

Both the treatment groups received conservative treatment, including education on knee OA, such as weight reduction, exercise, and lifestyle modification. Helpful activities were advised in order to reduce loading to the knee joint, for example, avoiding deep knee bending, low-seated activity, and climbing up and down stairs. The participants also received a brochure containing basic knowledge about knee OA.

According to the home-based exercise program, all participants were instructed by the physiotherapists on how to perform exercises correctly. The exercise program consisted of two strengthening exercises, including isometric and isotonic exercises of the quadriceps muscles. Each exercise was done ten times per set, for a total of two sets per day.

To check exercise compliance, the participants received an exercise logbook at their first visit of the treatment. They were oriented and assigned to record their daily exercise in this logbook. The frequency, type, and duration of the exercise were recorded and reviewed by the same assessor during each treatment session. Any adverse events from the laser treatment were also recorded, including eye injury, redness or heated skin, petechiae, or itching.

### 2.3. Measures

**Figure 1:**
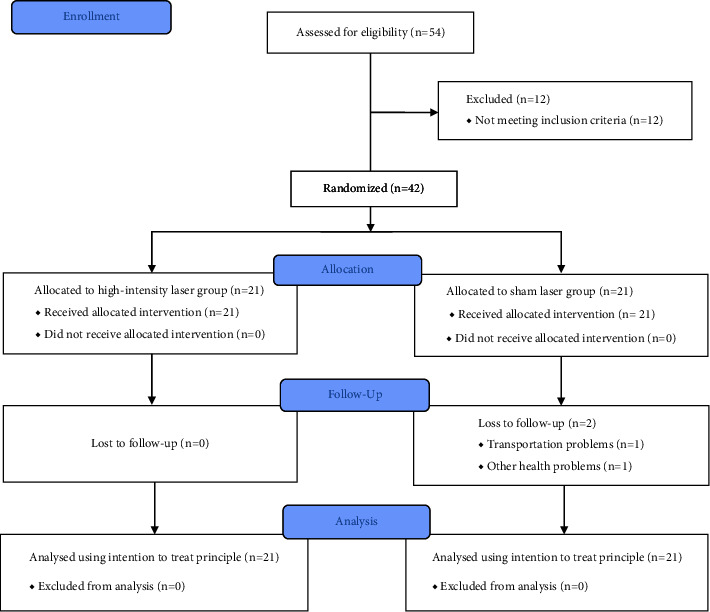
Consort diagram of the study protocol.

In this study, participants were randomly allocated to two groups, mainly the HILT group and the control group (21 participants per group), as shown in the consort diagram (Figure 1). The primary outcome was the pain score assessed using VAS, and the secondary outcome was score in the modified Thai version of Western Ontario and McMaster Universities Osteoarthritis Index (T-WOMAC). The two outcome measures were recorded at baseline and at the end of the study, or immediately after completion of the treatment. The VAS score was rated, with an overall score ranging from 1 to 10. A lower score indicates less pain, whereas a higher score indicates more severe pain. To rate the VAS score, the participants determined their pain level by crossing over a 10-cm line related to their pain perception. For clinical significance, the minimal clinically important difference (MCID) was a reduction in pain score by 30.0 mm [29].

The Western Ontario and McMaster Universities Osteoarthritis Index (WOMAC) was used to evaluate the functional outcomes in patients with knee OA [30]. The original WOMAC was divided into three categories of 24 subsections: pain (five subsections), joint stiffness (two subsections), and difficulty in performing daily activities or functional category (17 subsections) [31]. The total WOMAC score was 96 points (Likert scale) or 240 points (numerical rating scale). In this study, T-WOMAC was used to assess Thai participants. Unlike the original WOMAC, T-WOMAC consists of 22 subsections by removing F05 (bending to floor) and F12 (lying in bed) in the functional category. In addition, in this version, some questions in the functional category have been modified, such as items F09 (putting on socks), F11 (taking off socks), and F13 (getting in and out of the bathtub) to align with the culture and lifestyle of Thai people. The score for each item ranges from 0 to 10 points, with a score of 0 indicating task performance without difficulty, and a score of 10 indicating task performance with effort. Thus, the maximum score of T-WOMAC is 220 points. A previous study showed good validity and reliability of the T-WOMAC [32].

### 2.4. Statistical Analysis

All data were anonymous and analysed using R programming (https://www.r-project.org/) by a data analyst blinded to the treatment group allocation and outcome assessment. The normality of the two outcome measures was assessed using the Shapiro–Wilk test. Owing to the mixing of data between normal and nonnormal distributions, nonparametric statistical analysis was performed in this study. The Wilcoxon signed-rank test was used to evaluate changes within the same group, and the Mann–Whitney *U* test was used to compare differences between the two groups for both outcome measures.

Statistical significance was set at a *p* value of less than 0.05.

### 2.5. Sample Size Calculation

Sample size was estimated based on 80% power and a 2-tailed significant level of 0.05 in order to detect a 20% change in VAS, with a standard deviation (SD) of 2 points. Assuming a possible dropout rate of at least 10%, a total of 42 participants were recruited (21 participants per group).

## 3. Results

A total of 42 participants were recruited for the study (21 participants in each group). Two participants in the control group were lost to follow-up due to problems with transportation and other health problems that were not related to the adverse effects of the laser treatment. For those who were lost to follow-up, VAS and T-WOMAC were collected by phone. The remaining 40 participants received the HILT or sham laser for 10 sessions (Figure 1).

Table 1 shows the baseline characteristics of all individuals. The mean age (SD) of participants was 65.5 (8.8) years. Based on the severity of the disease, the numbers of participants in KL 2, 3, and 4 were 14, 17, and 11 people, respectively. All baseline variables, including the VAS and T-WOMAC scores, were not significantly different between the groups.

**Figure 2:**
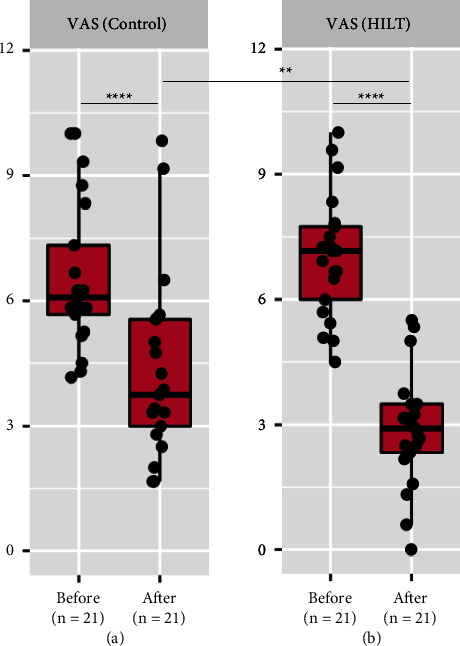
Box plot showing overall analysis of changes in the VAS scores within the same group and compared between the groups. (a). Change in the VAS score in the control group compared before and after treatment completion. (b). Change in the VAS score in the HILT group compared before and after treatment completion. Data are shown as ns (not significant) = *p* value >0.05, *∗* = *p* value <0.05, *∗∗* = *p* value <0.01, *∗∗∗* = *p* value <0.001, and *∗∗∗∗* = *p* value <0.0001. HILT, high-intensity laser therapy; VAS, visual analogue scale.

With regard to the primary outcome (VAS), overall, both the groups showed a statistically significant decline in the VAS score within the same group after completion of the treatment (*p* value <0.0001), in which the HILT group showed a greater reduction in the VAS score than the control group, as shown in the box plot. Moreover, a statistically significant difference between the groups was found, favouring HILT (*p* value <0.01) (Figures 2(a) and 2(b)).

**Figure 3:**
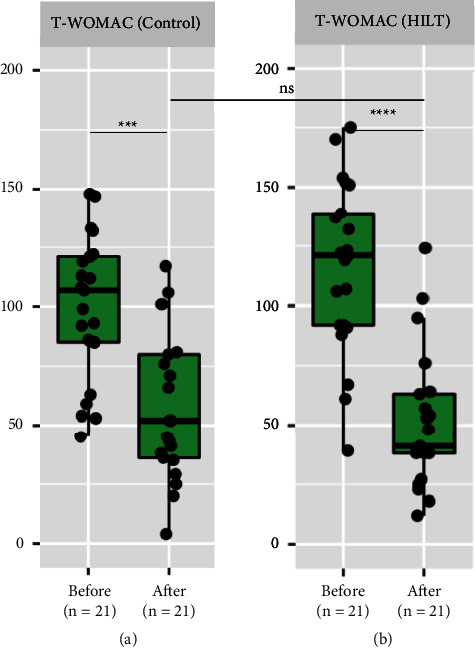
Box plot showing overall analysis of changes in the T-WOMAC scores within the same group and compared between the groups. (a). Change in the T-WOMAC score in the control group compared before and after treatment completion. (b). Change in the T-WOMAC score in the HILT group compared before and after treatment completion. Data are shown as ns (not significant) = *p* value >0.05, *∗* = *p* value <0.05, *∗∗* = *p* value <0.01, *∗∗∗* = *p* value <0.001, and *∗∗∗∗* = *p* value <0.0001. HILT, high-intensity laser therapy; T-WOMAC, the modified Thai version of Western Ontario and McMaster Universities Osteoarthritis Index.

Regarding the secondary outcome (T-WOMAC), the T-WOMAC score decreased significantly in both the groups at the end of the study (*p* value <0.001), with a more significant change in the HILT group. However, between-group comparisons did not show statistically significant differences (Figures 3(a) and 3(b)). For more details on the overall changes in the two outcomes, please see Supplementary Table S1.

Exercise compliance was excellent in both the groups, as assessed by data from the exercise logbook. The intervention group showed an adherence to exercise of about 95.4%, with the control group at 92.0%. There was no significant difference in exercise compliance between the groups. Apart from this, no adverse events were reported during the study period.

## 4. Discussion

The present study was set as a single-blind, randomised controlled trial comparing the efficacy of the HILT to that of sham laser plus conservative treatment for short-term pain reduction in patients with various degrees of knee OA. At the end of the study, within the same group, there was a statistically significant reduction in the VAS and T-WOMAC scores in both the intervention and control groups. Between-group comparisons also revealed a significant reduction in the VAS score, favouring HILT. Importantly, the overall change in VAS in the HILT group showed not only statistical but also clinical significance, in which VAS was reduced by more than 30.0 mm from the baseline.

### 4.1. Pain

In terms of pain reduction, the main findings of this study were consistent with previous studies [20–23, 33, 34]. These studies showed significant pain reduction after 7 days to 3 months of treatment with HILT.

Despite the fact that the overall findings of the present study revealed a significant reduction in VAS, there were differences between the present study and previous studies, mainly in terms of the participants and the HILT treatment protocol. First, the participants in this study had any stage of knee OA, including moderate-to-severe knee OA (KL 3–4), with approximately two-thirds of the study population (66.7 percent), which was more severe than the studies conducted in the previous reports (mainly KL 2–3). Second, the total energy of laser treatment in this study was 562.5 J per session, which was lower than most of the previous investigations, which used laser energy ranging from 1250–3000 J per session [20–23, 34]. We discovered that the lower dosage protocol also resulted in significant pain reduction in patients with knee OA who had KL 2–4. This could imply that the lower-dose HILT protocol would be as effective as the higher-dose protocol for pain reduction. However, the duration of treatment for the lower-dose regimen would be shorter while still providing effective pain control.

The analgesic effects of HILT are thought to be due to several mechanisms. The first is the modulation of pain. Laser helps in promoting the release of endogenous opioids such as beta-endorphins and serotonin at the peripheral nerves where nociceptors are located [35, 36]. Once endogenous opioid levels increase, they bind to nociceptors, thereby occupying the binding sites from external noxious stimuli. Second, laser treatment reduces ATP production and calcium influx to the dorsal root ganglion neurons and increased intracellular reactive oxygen species, disrupting the propagation of pain action potential and resulting in pain attenuation [37, 38].

In addition to the mechanisms mentioned above, the other two important components of the analgesic effects of HILT are gate control theory and nerve fibre regeneration [20, 22]. In addition, photochemical and photothermic effects of laser might help stimulate blood flow and cell metabolism, as well as promote Schwann cell proliferation, nerve fibre regeneration, and nearby collateral sprouting [39]. HILT not only promotes analgesic effects but also decreases inflammation. There is evidence that lasers could help decrease proinflammatory cytokines as well as inflammatory mediators, such as interleukin-1, interleukin-6, prostaglandin, C-reactive protein, and tumour necrosis factor-alpha [16].

Not only for its analgesic and anti-inflammatory effects, HILT also promotes bio-stimulation in knee OA. Alayat et al. [34] investigated the effects of HILT twice per week for 6 weeks. They found a significant increase in synovial thickness in the HILT group compared with the other two groups receiving glucosamine plus exercise and placebo laser plus exercise. Furthermore, a recent study by Alkatan et al. [33] also showed a significant increase in femoral cartilage thickness of the knee joint in the HILT group after 6 weeks of treatment. Thus, HILT could ameliorate intraarticular cartilage loss which is one of the key pathophysiologies of knee OA. However, the present study did not measure outcomes, such as sonography, which reflects the bio-stimulating effect of HILT.

### 4.2. The Modified Thai Version of Western Ontario and McMaster Universities' Osteoarthritis Index

Regarding the secondary outcome, we expected that significant pain reduction would have positive effects on functional improvement in patients with knee OA, as measured by the T-WOMAC score. Previous studies showed a significant improvement in WOMAC score after 4–12 weeks of treatment with HILT [21–23, 34]. However, our research found that T-WOMAC scores decreased significantly only within the same group, but not between the two. As a result, the findings of this trial could not infer that HILT improved function in patients with knee OA more than a sham laser. We presumed that the nonsignificant difference between the T-WOMAC groups in this study could be explained by several factors. One factor is the degree of the disease severity; as mentioned above, two-thirds of the participants in this study had moderate-to-severe knee OA. Therefore, a greater disease severity might have a negative impact on baseline patients' conditioning as well as functional status, compared to a less severe degree. Another possible factor might be due to the exercise program. In our study, the exercise program focused only on quadriceps muscle strength. Other types of exercise, such as aerobic exercise, to improve general conditioning were not included, which could have contributed to the nonsignificant difference in T-WOMAC scores between the two groups. Furthermore, the study was conducted over a short period of time, which may have been inadequate for regaining muscle strength, particularly in patients who had deconditioning. The last reason could be the laser treatment protocol. The current study used a lower dosage of HILT per session compared to earlier studies. This treatment protocol may be insufficient to provide a significant change between the two groups in terms of functional outcomes.

The present study has some limitations. First, regarding the blinding of the study, the outcome assessor and the physical therapists who provided treatment were aware of the treatment group allocation, which might have led to study bias. In this study, however, study blinding was performed on the participants and data analyst. Second, the WOMAC subscales of pain, joint stiffness, and function were not analysed; therefore, we did not know whether function or joint stiffness subscales would have statistically significant changes after HILT. Additionally, due to the modification of some items in the T-WOMAC, the comparison of the T-WOMAC in the present study to the original WOMAC in the others might be considered. Third, to determine the true effects of HILT in patients with severe knee OA, further studies should either stratify HILT effects based on the disease severity or recruit only participants with KL 4 or severe degree. Fourth, the laser treatment protocol in this study was performed in a range of 2–3 sessions per week or 10 sessions in 4–5 weeks. Thus, we did not know whether 2 or 3 sessions per week could have better results for pain reduction. Nevertheless, previous studies [21–23, 34] that applied HILT for 2–3 sessions per week for a total of 12 sessions showed a statistically significant reduction in the VAS score after HILT. Furthermore, in this study, we used the lower-dose HILT protocol, compared to the previous studies. To evaluate the efficacy of the lower dosage regimen, a further study comparing the effects of low and high doses of the HILT protocol should be conducted. Lastly, the present study did not evaluate the long-term effects of HILT. Thus, future research is needed to determine the long-term effects of HILT, not only on pain score and function but also on other patient-related outcomes.

## 5. Conclusion

Our research findings indicate that HILT is still an effective treatment for osteoarthritis knee pain, even when provided at a lower dosage. Although there have been previously reported similar results to this study, the present study also supports this positive effect of HILT on pain relief in a different cohort population and with a different laser treatment protocol. The results from our study might be implemented in clinical practice such that a lower-dose protocol of HILT could promote short-term pain relief in any stage of knee OA. The advantage of this treatment protocol is that it may provide effective pain reduction in a shorter treatment time with fewer adverse effects than the high-dose protocol. This would be beneficial, especially for those who refuse surgical treatment or high-risk patients contraindicated for surgery due to reasons such as multiple comorbidities or older people.

## Figures and Tables

**Table 1:** Baseline demographic and clinical characteristics of the participants.

Baseline characteristics	HILT (*n* = 21)	Control (*n* = 21)	*p* value
Mean age (SD), years	66.1 (9.4)	65.0 (8.5)	0.668
BMI. mean (SD), kg/m^2^	28.1 (5.2)	27.4 (5.8)	0.676

*Sex, number of patients (%)*			
Female	18 (85.7)	16 (76.2)	0.697
Male	3 (14.3)	5 (23.8)	
KOA severity (KL classification),			0.559

*Number of patients (%)*			
2	6 (28.6)	8 (38.1)	
3	8 (38.1)	9 (42.9)	
4	7 (33.3)	4 (19.0)	
VAS, mean (SD)	7.02 (1.47)	6.54 (1.76)	0.349
T-WOMAC, mean (SD)	116.09 (35.92)	99.57 (31.04)	0.119

BMI, body mass index; HILT, high-intensity laser therapy; KL, Kellgren and Lawrence; T-WOMAC, the modified Thai version of Western Ontario and McMaster Universities Osteoarthritis Index; VAS, visual analogue scale.

## Data Availability

The data used to support the findings of the study are available upon request from the corresponding author.

## Supplementary Materials

Supplementary Materials Supplementary Table 1. Overall changes in the VAS and T-WOMAC scores within the same group and between the two groups. Supplementary Figure 1. The laser-irradiated points.

## References

[1] Hunter D. J., McDougall J. J., Keefe F. J. (2008). The symptoms of osteoarthritis and the genesis of pain. *Rheumatic Disease Clinics of North America*.

[2] Jang S., Lee K., Ju J. H. (2021). Recent updates of diagnosis, pathophysiology, and treatment on osteoarthritis of the knee. *International Journal of Molecular Sciences*.

[3] Thakur M., Dickenson A. H., Baron R. (2014). Osteoarthritis pain: nociceptive or neuropathic?. *Nature Reviews Rheumatology*.

[4] Park H. M., Kim H. S., Lee Y. J. (2020). Knee osteoarthritis and its association with mental health and health-related quality of life: a nationwide cross-sectional study. *Geriatrics and Gerontology International*.

[5] Törmälehto S., Mononen M. E., Aarnio E., Arokoski J. P. A., Korhonen R. K., Martikainen J. (2018). Health-related quality of life in relation to symptomatic and radiographic definitions of knee osteoarthritis: data from Osteoarthritis Initiative (OAI) 4-year follow-up study. *Health and Quality of Life Outcomes*.

[6] Cleveland R. J., Alvarez C., Schwartz T. A. (2019). The impact of painful knee osteoarthritis on mortality: a community-based cohort study with over 24 years of follow-up. *Osteoarthritis and Cartilage*.

[7] Cui A., Li H., Wang D., Zhong J., Chen Y., Lu H. (2020). Global, regional prevalence, incidence and risk factors of knee osteoarthritis in population-based studies. *EClinicalMedicine*.

[8] Gianola S., Bargeri S., Del Castillo G. (2022). Effectiveness of treatments for acute and subacute mechanical non-specific low back pain: a systematic review with network meta-analysis. *British Journal of Sports Medicine*.

[9] Maghbool M., Khosravi T., Vojdani S. (2020). The effects of eugenol nanoemulsion on pain caused by arteriovenous fistula cannulation in hemodialysis patients: a randomized double-blinded controlled cross-over trial. *Complementary Therapies in Medicine*.

[10] Bannuru R. R., Osani M. C., Vaysbrot E. E. (2019). OARSI guidelines for the non-surgical management of knee, hip, and polyarticular osteoarthritis. *Osteoarthritis and Cartilage*.

[11] Jevsevar D. S., Brown G. A., Jones D. L. (2013). The American Academy of Orthopaedic Surgeons evidence-based guideline on: treatment of osteoarthritis of the knee. *The Journal of Bone & Joint Surgery*.

[12] Kolasinski S. L., Neogi T., Hochberg M. C. (2020). 2019 American College of rheumatology/arthritis foundation guideline for the management of osteoarthritis of the hand, hip, and knee. *Arthritis Care & Research*.

[13] Mester A. (2013). Laser biostimulation. *Photomedicine and Laser Surgery*.

[14] Tomazoni S. S., Leal-Junior E. C. P., Pallotta R. C., Teixeira S., de Almeida P., Lopes-Martins R. Á. B. (2017). Effects of photobiomodulation therapy, pharmacological therapy, and physical exercise as single and/or combined treatment on the inflammatory response induced by experimental osteoarthritis. *Lasers in Medical Science*.

[15] Xiang A., Deng H., Cheng K. (2020). Laser photobiomodulation for cartilage defect in animal models of knee osteoarthritis: a systematic review and meta-analysis. *Lasers in Medical Science*.

[16] Pallotta R. C., Bjordal J. M., Frigo L. (2012). Infrared (810-nm) low-level laser therapy on rat experimental knee inflammation. *Lasers in Medical Science*.

[17] Wang P., Liu C., Yang X. (2014). Effects of low-level laser therapy on joint pain, synovitis, anabolic, and catabolic factors in a progressive osteoarthritis rabbit model. *Lasers in Medical Science*.

[18] Stausholm M. B., Naterstad I. F., Joensen J. (2019). Efficacy of low-level laser therapy on pain and disability in knee osteoarthritis: systematic review and meta-analysis of randomised placebo-controlled trials. *BMJ Open*.

[19] Ezzati K., Laakso E. L., Salari A., Hasannejad A., Fekrazad R., Aris A. (2020). The beneficial effects of high-intensity laser therapy and Co-interventions on musculoskeletal pain management: a systematic review. *Journal of Lasers in Medical Sciences*.

[20] Angelova A., Ilieva E. M. (2016). Effectiveness of high intensity laser therapy for reduction of pain in knee osteoarthritis. *Pain Research and Management*.

[21] Kim G. J., Choi J., Lee S., Jeon C., Lee K. (2016). The effects of high intensity laser therapy on pain and function in patients with knee osteoarthritis. *Journal of Physical Therapy Science*.

[22] Nazari A., Moezy A., Nejati P., Mazaherinezhad A. (2019). Efficacy of high-intensity laser therapy in comparison with conventional physiotherapy and exercise therapy on pain and function of patients with knee osteoarthritis: a randomized controlled trial with 12-week follow up. *Lasers in Medical Science*.

[23] Kheshie A. R., Alayat M. S. M., Ali M. M. E. (2014). High-intensity versus low-level laser therapy in the treatment of patients with knee osteoarthritis: a randomized controlled trial. *Lasers in Medical Science*.

[24] Song H. J., Seo H. J., Kim D. (2020). Effectiveness of high-intensity laser therapy in the management of patients with knee osteoarthritis: a systematic review and meta-analysis of randomized controlled trials. *Journal of Back and Musculoskeletal Rehabilitation*.

[25] Kohn M. D., Sassoon A. A., Fernando N. D. (2016). Classifications in brief: kellgren-lawrence classification of osteoarthritis. *Clinical Orthopaedics and Related Research*.

[26] Hegedus B., Viharos L., Gervain M., Gálfi M. (2009). The effect of low-level laser in knee osteoarthritis: a double-blind, randomized, placebo-controlled trial. *Photomedicine and Laser Surgery*.

[27] WALT (2010). Recommended treatment doses for low level laser therapy. https://waltpbm.org/documentation-links/recommendations/.

[28] Bjordal J. M., Couppé C., Chow R. T., Tunér J., Ljunggren E. A. (2003). A systematic review of low level laser therapy with location-specific doses for pain from chronic joint disorders. *Australian Journal of Physiotherapy*.

[29] Lee J. S., Hobden E., Stiell I. G., Wells G. A. (2003). Clinically important change in the visual analog scale after adequate pain control. *Academic Emergency Medicine*.

[30] Bellamy N., Buchanan W. W., Goldsmith C. H., Campbell J., Stitt L. W. (1988). Validation study of WOMAC: a health status instrument for measuring clinically important patient relevant outcomes to antirheumatic drug therapy in patients with osteoarthritis of the hip or knee. *Journal of Rheumatology*.

[31] Bellamy N. (1995). WOMAC Osteoarthritis Index: A User’s Guide. *Victoria Hospital*.

[32] Kuptniratsaikul V., Rattanachaiyanont M. (2007). Validation of a modified Thai version of the Western Ontario and McMaster (WOMAC) osteoarthritis index for knee osteoarthritis. *Clinical Rheumatology*.

[33] Akaltun M. S., Altindag O., Turan N., Gursoy S., Gur A. (2021). Efficacy of high intensity laser therapy in knee osteoarthritis: a double-blind controlled randomized study. *Clinical Rheumatology*.

[34] Alayat M. S. M., Aly T. H. A., Elsayed A. E. M., Fadil A. S. M. (2017). Efficacy of pulsed Nd: YAG laser in the treatment of patients with knee osteoarthritis: a randomized controlled trial. *Lasers in Medical Science*.

[35] Hagiwara S., Iwasaka H., Hasegawa A., Noguchi T. (2008). Pre-Irradiation of blood by gallium aluminum arsenide (830 nm) low-level laser enhances peripheral endogenous opioid analgesia in rats. *Anesthesia & Analgesia*.

[36] Navratil L. H., Dylevsky I. (1997). Mechanisms of the analgesic effect of therapeutic lasers IN VIVO. *Laser Therapy*.

[37] Chow R., Armati P., Laakso E. L., Bjordal J. M., Baxter G. D. (2011). Inhibitory effects of laser irradiation on peripheral mammalian nerves and relevance to analgesic effects: a systematic review. *Photomedicine and Laser Surgery*.

[38] Zupin L., Ottaviani G., Rupel K. (2019). Analgesic effect of Photobiomodulation Therapy: an in vitro and in vivo study. *Journal of Biophotonics*.

[39] de Oliveira R. F., de Andrade Salgado D. M. R., Trevelin L. T. (2015). Benefits of laser phototherapy on nerve repair. *Lasers in Medical Science*.

