# Supplementary material for: Short-Term Efficacy of High-Intensity Laser Therapy in Alleviating Pain in Patients with Knee Osteoarthritis: A Single-Blind Randomised Controlled Trial
Source: Pain Res Manag. 2022 Oct 21;2022:1319165. doi: 10.1155/2022/1319165 (PMC9616657; doi:10.1155/2022/1319165)
Supplement: Supplementary Materials — Supplementary Table 1. Overall changes in the VAS and T-WOMAC scores within the same group and between the two groups. Supplementary Figure 1. The laser-irradiated points. [file 1319165.f1.docx]

**Supplementary data**

**Table S1.** **Overall changes in VAS and T-WOMAC scores within the same group and between the two groups**

|  | Pre-treatment | Post-treatment | Mean difference (95%CI) | *p-*value |
| --- | --- | --- | --- | --- |
| VAS, mean (SD) |  |  |  |  |
| HILT | 7.02 (1.47)  (n=21) | 2.88 (1.38)  (n=21) | 4.14  (3.45-4.82) | <0.0001^b^ |
| Control | 6.54 (1.76)  (n=21) | 4.33 (2.18)  (n=19) | 2.21  (1.31-3.12) | <0.0001^b^ |
| *p*-value | 0.349^c^ | 0.017^a^ | 0.002^a^ |  |
| T-WOMAC, mean (SD) |  |  |  |  |
| HILT | 116.09 (35.92)  (n=21) | 51.29 (28.69)  (n=21) | 64.81  (50.40-79.22) | <0.0001^b^ |
| Control | 99.57 (31.04)  (n=21) | 58.05 (31.23)  (n=19) | 41.52  (25.57-57.47) | <0.001^b^ |
| *p-*value | 0.119^c^ | 0.48^c^ | 0.056^c^ |  |

HILT, high-intensity laser therapy; T-WOMAC, the modified Thai version of Western Ontario and McMaster Universities Osteoarthritis index; VAS*,* visual analogue scale.

^a^Significant difference between the groups

^b^Significant change within the same group

^c^No significant difference


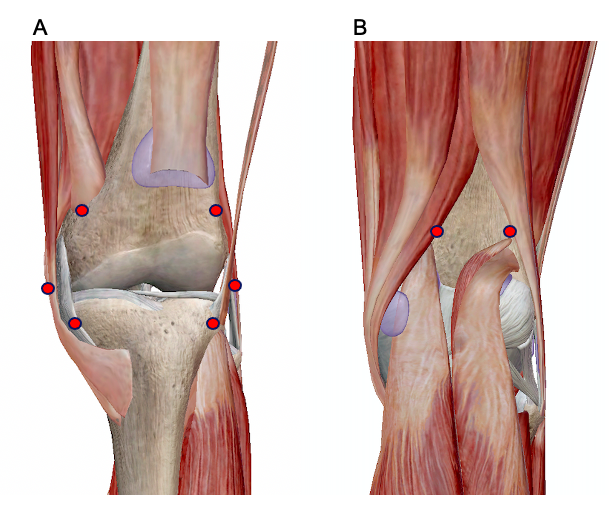


**Figure S1.** **The figure shows eight points of laser treatment.** A. Six points at the medial and lateral epicondyle of the femur, medial and lateral condyle of the tibia, and medial and lateral knee gap, where the laser was applied on the anterior knee in the supine position with 30 ° of knee flexion. B. Two points at the medial edge of the biceps femoris and semitendinosus tendon, where the laser was applied on the posterior knee in the prone position with the knee fully extended.

(Note: The figure was created by Visible Body application.)
